# Formation and Emissions of Volatile Organic Compounds from Homo-PP and Co-PP Resins during Manufacturing Process and Accelerated Photoaging Degradation

**DOI:** 10.3390/molecules25122761

**Published:** 2020-06-15

**Authors:** Peng Kang, Peng Wu, Yan Jin, Shengpeng Shi, Dali Gao, Guangxin Chen, Qifang Li

**Affiliations:** 1 State Key Laboratory of Chemical Resource Engineering, Beijing University of Chemical Technology, Beijing 100029, China; kangpeng.bjhy@sinopec.com (P.K.); wupeng.bjhy@sinopec.com (P.W.); gxchen@mail.buct.edu.cn (G.C.); 2 Polymer Processing R&D Division, Sinopec Beijing Research Institute of Chemical Industry, Beijing 100013, China; jiny1902@126.com (Y.J.); shisp.bjhy@sinopec.com (S.S.); gaodl.bjhy@sinopec.com (D.G.); 3 College of Materials Science and Engineering, Beijing University of Chemical Technology, Beijing 100029, China

**Keywords:** PP resin, photo oxidation, volatile organic compounds, HS-GC-FID/MS, degradation, molecular structure

## Abstract

Volatile organic compounds (VOCs) from polypropylene (PP) seriously restricts the application of PP in an automotive field. Herein, the traceability of VOCs from PP resins during manufacturing process and accelerated photoaging degradation was clarified on basis of an accurate characterization method of key VOCs. The influence of PP structures on changing the accelerated photoaging degradation on the VOCs was systematic. The VOCs were identified by means of Gas chromatography (GC) coupled with both a hydrogen flame ion detector (FID) and a mass spectrometry detector (MSD). Results showed that both the molecular structure of PP and the manufacturing process affected the species and contents of VOCs. In addition, the photoaging degradation of PP resulted in a large number of new emerged volatile carbonyl compounds. Our work proposed a possible VOC formation mechanism during the manufacturing and photoaging process. VOCs from PP resins were originated from oligomers and chain random scission during thermomechanical degradation. However, β scission of alkoxy radical and Norrish tape I reactions of ketones via intermediate transition were probably the main VOCs formation routes towards PP during photoaging degradation. This work could provide scientific knowledge on both the accurate traceability of VOCs emissions and new technology for development of low-VOCs PP composites for vehicle.

## 1. Introduction

Polypropylene (PP), due to the excellent cost-performance ratio and material versatile properties superior to those of other polyolefins [1,2,3,4,5], is a kind of commodity plastic widely used in automotive, packaging, construction, wire and cable, mechanical engineering, electric and electronic industries and other industrial fields [6,7,8,9,10]. PP has become the most important thermoplastic material in the automobile industry [11]. PP homopolymers, random copolymers and impact copolymers are used in products such as automotive parts and battery cases, carpeting, electrical insulation and fabrics [12]. Moreover, the application of PP in the automobile market is the largest [13]. However, its application is seriously restricted by Volatile Organic Compounds (VOCs) emissions, which worsen the indoor air quality and consequently pose a threat to human health [14,15,16,17,18]. Therefore, the VOCs emission is an important index for the interior materials used as automobile parts [15].

An amount of attentions has been paid on clarifying the mechanism in the VOCs emission from PP composites [19,20,21,22,23]. Several studies have pointed out that VOCs emission was generated from the random chain scissions of PP main chains [24] or/and the oxidation reaction of tertiary carbon atom of PP during the melt-mixing processing [25,26,27,28], as well as residual catalysts and antioxidant additives. Some other studies have found that thermal degradation of PP at about 300–600 °C, which was much higher than the processing temperature of PP resin, also generated lots of VOCs [22,29,30]. However, the distribution of VOCs species and amounts from PP composites with various formula is quite different and the corresponding source of VOCs is quite different [22]. It can be speculated that PP matrix resin may have a great impact on the VOCs emissions of PP composites but there is less research on VOCs of different kinds of PP resins. PP can be divided into homo-PP and co-PP according to its molecular structure [31]. In addition, the different manufacturing process [32] could also lead to PP with different structures, for example, the impact co-PP manufactured by the peroxide degradation method or the hydrogen regulation method would probably result in the variety of VOCs emissions.

The interior materials in the automobiles contain PP, Polyethylene (PE), Acrylonitrile Butadiene Styrene copolymer (ABS) and polyurethane (PU) and so forth, which will inevitably suffer from sunlight during the use of the automobile [33,34,35]. Therefore, the influence of photoaging on VOCs is very important. Some literatures [36,37,38,39] have analyzed the photoaging degradation products of PP during UV, γ irradiation and so forth by means of Fourier Transform Infrared Spectroscopy (FTIR), Mass Spectroscopy (MS) or Gas Chromatography-Mass Spectroscopy (GC-MS) analysis. Most of these studies only studied the VOCs remain in photoaged PP samples but lack research on VOCs migration from the PP solid samples to the environment during photoaging. Additionally, some researchers utilized the irradiation for a limited test period but ignored adaptation for the automobile field, in which the xenon lamp is usually used to simulate sunlight. Therefore, photoaging tests based on xenon lamp conditions may have more potential application value for the automobile industry.

Herein, commercial homo-PP and impact co-PP neat resins with three grades having the similar Melt Flow Rate (MFR) were chose as representatives to study VOCs emissions during the manufacturing process and accelerated photo aging in the Xenon lamp aging chamber. VOCs generated from PP resins during different photoaging conditions were collected in real time and then variations of the species and relative proportions of VOCs were tracked by Headspace extraction-Gas Chromatography-Hydrogen Flame ion Detector/Mass Spectroscopy (HS-GC-FID/MS). Furthermore, the changes of structures of photoaged PP, including the molecular weight, molecular weight distribution, chemical functional groups and crystallites structure, were studied using GPC (Gel Permeation Chromatography), FTIR and DSC (Differential Scanning Calorimetry) in detail. The effects of the molecular structure, manufacturing process and accelerated photoaging of PP resins on VOCs emissions from PP resins were consequently illustrated. Finally, the VOCs formation mechanism during manufacturing and photoaging process was proposed. Results could provide the scientific knowledge for both the accurate traceability of VOCs emissions and the new technology for development of low-VOCs PP composites for vehicles.

## 2. Results and Discussion

### 2.1. VOCs Emissions from PP Resins without Photoaging

**Figure 1:**
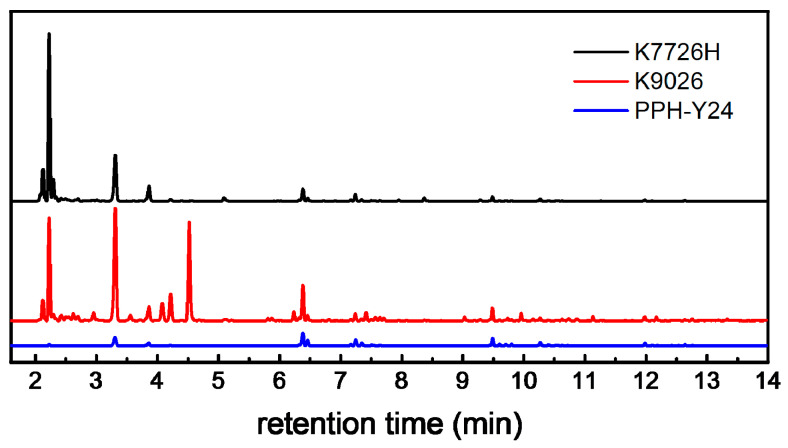
Gas Chromatography-Hydrogen Flame Ion Detector (GC-FID) chromatograms showing volatile organic compounds (VOCs) extracted by Headspace (HS) from neat PP resins (K7726H: the black line, K9026: the red line, PPH-Y24: the blue line).

The VOCs emissions from neat PP resins were clearly identified by HS-GC-FID/MS as shown in Figure 1.

There are 47, 43 and 64 identified chromatographic peaks ascribed to VOCs from PPH-Y24, K7726H and K9026, respectively. It shows that the neat PP resins contain more than 40 species of VOCs, which were generated by the manufacturing process. Moreover, there are obviously a different number and intensities of chromatographic peaks among VOCs from different PP resins. It indicated that the PP resins with various parameters such as the catalysts system, the polymerization process, the pelleting temperature which could approach to 230 °C at the maximum and the molecular weight distribution seemed to influence the VOCs from PP resins. PPH-Y24 emitted the least species of VOCs in the least abundances, which was vividly shown in GC chromatograms (Figure 1). On the contrary, more species and amounts of VOCs were released from both K7726H and K9026. In addition, their abundances were much higher than those from PPH-Y24. It indicated that both the species and amounts of VOCs from co-PP were higher than those from homo-PP. Therefore, the addition of ethylene in polymerization could reduce the aging stability of PP [40] and further infect the VOC formation. The potential compounds identification of VOCs was carried out with NIST mass spectral library and according to the published literature [36,37,38]. The most relevant VOCs with the large peak area were identified and listed in Table 1.

Some of the VOCs, which originated from the molecules containing more than nine carbon atoms, are various isomers. Because of the limited accuracy of mass spectrometry itself, some alkanes isomers with side groups can only identify to aliphatic hydrocarbon but difficultly determine their chemical formulas as well as molecular structural formulas. However, the identification of precise chemical structure formula was obtained by the related literature and the subsequent analysis of VOCs emission mechanism from PP resin. Thus, some VOCs isomers with different retention time on gas chromatography but probably identical molecular formulas should be understood.

The notable differences among PP resins on VOCs are some compounds up to C_12_, whereas there are only slight differences on VOCs above C_12_ which are difficult to evaporate due to their high boiling points. As shown in Table 1 (“0 h” column), aliphatic hydrocarbons are present in all PP resins. However, VOCs with oxygen groups were only present in co-PP K7726H and K9026, which may arise from the ethylene-propylene copolymerization. Firstly, aliphatic hydrocarbons having a characteristic of a multiplier of three carbon atoms, such as C_6_H_14_, C_9_H_20_, C_12_H_26_, C_15_H_32_ and C_18_H_38_, might come from either oligomers generated during the PP polymerization because of multiple active sites of the catalyst or thermomechanical degradation products in pelleting. In addition, some VOCs with oxygen-containing groups such as *n*-butanol, *t*-butanol, acetone, ethanol and so forth, might be generated from thermo-oxidation degradation of PP during the processing, the peroxide degradation of PP chain which were dependent on different manufacturing processes. Moreover, aldehydes and ketones were only present in VOCs of K9026, probably attributing to its unique manufacturing process.

Since PPH-Y24 and K7726H were both produced by the hydrogen regulation method, compare VOCs of PPH-Y24 with those of K7726H could provide reliable information about the effect of molecular structure on VOCs emission. VOCs with oxygen-containing group were present in both K7726H and PPH-Y24 (Table 1). It was probably due to thermo-oxidation of PP resins during manufacturing processing. However, it was necessary to add small amounts of O_2_ and H_2_O artificially during the copolymerization stage just for adjusting and inactivating the catalyst activity, respectively. Besides, the hydrolysis of the catalyst can also produce some alcohols. Moreover, the concentration of O_2_ which was added only in copolymerization can be ignored because of a great amount N_2_ and there were no oxidation of PP in the twin-screw extruder. Therefore, some alcohols were generated in the copolymerization process of co-PP, rather than in homo-PP. Besides, alcohols, the amounts of aliphatic hydrocarbon from co-PP were also much higher than those of homo-PP. This was also resulted from the different polymerization processes. The co-PP was manufactured by the two-step polymerization process including ethylene addition in the copolymerization stage to form the EPR rubber phase following the homo-polymerization of propylene with addition of hydrogen in the first step. In general, the molecular weight of the PP fraction in the co-PP decreased by the hydrogen termination process and this also resulted in the formation of VOCs. Besides, the EPR rubber fraction of the co-PP usually had a higher molecular weight, so that the MFR of PP fraction in co-PP should be higher than that of PPH-Y24 in order to have the final similar MFR values. Therefore, more PP chains in co-PP resin probably chain transfers reactions and would produce the higher amounts of VOCs. It is worth mentioning that there is insignificant difference for the species of VOC between homo-PP and co-PP. It indicated the aliphatic hydrocarbons was mainly generated from PP fraction rather than the rubber fraction in co-PP resin, however, the small amounts alcohols were produced in co-polymerization process.

As shown in Table 1, the manufacturing process had significant impacts on both the species and amounts of VOCs from two co-PP resins. K9026 manufactured by adding peroxide agent in pelleting emitted amounts of oxygen-containing compounds, such as acetone and tertbutyl alcohol, which might be generated from the decomposition of peroxides and PP. While, related oxygen-containing compounds were absent in K7726H manufactured by the hydrogen-regulating method, as shown vividly by the absences of peaks of acetone and tertbutyl alcohol. It was shown that the peroxide degradation method is the main factor to greatly increase the amount of VOCs. Instead, the amount of other compounds excluding acetone and tertbutyl alcohol from K9026 was rather lower than that of K7726H. It seemed that the VOCs generated from polymerization from K9026 were less than those from K7726H. It is mainly due to the lower MFR of PP powder of K9026 than that of PP powder of K7726H. The K9026 powder with low hydrogen addition in polymerization would generate small amount of VOCs. Therefore, the oxygen-containing compounds such as acetone and tertbutyl alcohol were generated more seriously by the peroxide degradation method than by the hydrogen regulation method, however, other VOCs from co-PP resins were the opposite.

Furthermore, there were more species and higher amount of VOCs emission from co-PP K9026 than that from homo-PP PPH-Y24. It might be attributed to both molecular structures and manufacturing processes. But, the effect of molecular structure on VOCs was less pronounced than that of manufacture process. Therefore, the ranking of the factors affecting the VOCs of PP resins was as follows: the effect of the peroxide degradation method was the largest, followed by the hydrogen regulation method and that of the molecular structure was the smallest.

**Figure 2:**
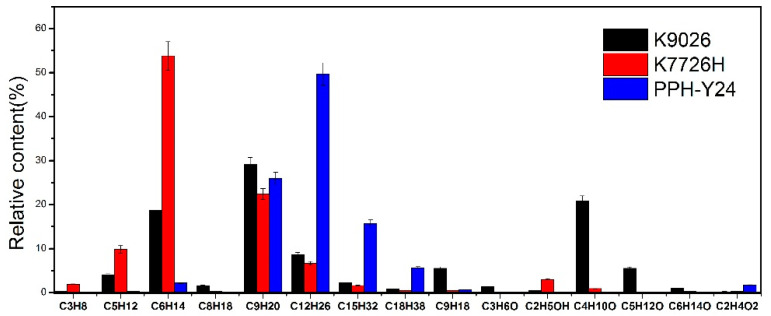
VOC species and relative content from different polypropylene resins.

Moreover, as shown in Figure 2, the relative proportions of VOCs were also affected by molecular structure and the manufacturing process of PP. The distribution of VOCs from PPH-Y24 contained alkanes (97.5 area %), small amounts of alkenes (0.58 area %) and acetic acid (1.73 area %). While, K7726H generated VOCs with the high contents of alkanes (97.0 area %) as well as small amounts of alkenes (0.5 area %) and alcohols (4.2 area %). Additionally, K9026 also produced VOCs with a major of alkanes (65.3 area %) as well as a few alkenes (5.5 area %), ketones and alcohols (29.0 area %). It was concluded that the distribution of VOCs from homo-PP and co-PP resin was dependent on the molecular structure and manufacturing process. Therefore, it can also provide a certain data reference for identifying different PP resins by tracing the distribution of VOCs.

### 2.2. Changes of VOCs Emission from PP Resins during the Accelerated Photoaging

**Figure 3:**
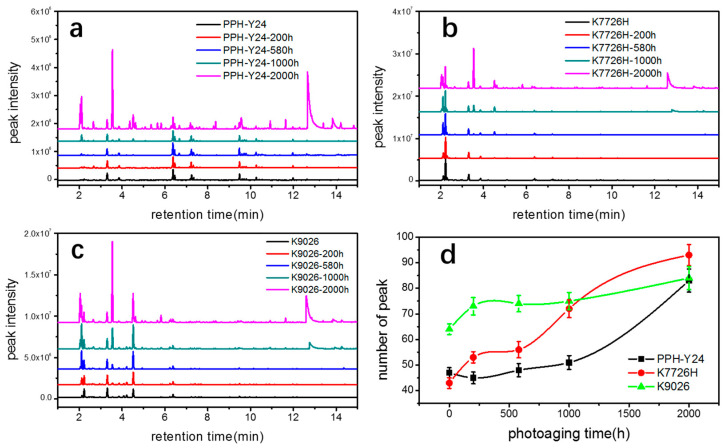
VOC chromatogram of three kinds of PP resin ((**a**) K7726H, (**b**) K9026, (**c**) PPH-Y24) and the number of chromatogram peak (**d**) with various accelerated photo aging exposure time.

Figure 3a–c shows chromatograms of VOCs from PP resins along with the photoaging time and the most relevant compounds identified are listed in Table 1. The number and intensities of chromatographic peaks of three PP resins increased with extending the photoaging time, indicating that accelerate photoaging resulted in the increase of both species and amounts of VOCs from three PP resins. Monitoring the evolution of the various degradation compounds upon the photoaging process is very different. Some compounds only appeared in the early stages of photo degradation but they did not display remarkable changes even in the last stages of the ageing process. And some compounds seemed to present after a period of photoaging; however, some compounds kept stable during all photoaging conditions.

As shown in Figure 3d, the photoaging resulted in a rapid increase of the number of peaks from 40~65 to 80~95. It showed that the photoaging led to a larger number of new emerged VOCs from three PP resins. The newly emerged VOCs included oxygen-containing compounds, such as carbonyl compounds, aldehydic compounds, alcohol compounds and carboxylic compounds, as well as aliphatic hydrocarbon compounds, for instance butene and isopentane. The amount of these newly emerged VOCs increased obviously with prolonging the photoaging time, indicating that the new emerged VOCs were products of photo oxidative degradation. The species and amounts of VOCs from all PP resins increased significantly during photoaging, however, the changes of VOCs with various responses to photoaging were further analyzed by molecular structure, aggregate structure and manufacturing process.

The comparison of chromatographic peak areas of all three PP resins with various photoaging time is presented in Table 1. It showed that the peak areas and peak numbers of PP resins when the photoaging of 200 h did not increase significantly, confirming that photoaging time of 200 h had little effect on the photoaged PP resins. When the photoaging time further increased to 500 h, 1000 h and 2000 h, the differences in the peak areas and peak numbers of PP resins became more and more obviously. The changes of VOCs from homo-PP PPH-Y24 were few during the period of 0 h to 1000 h of photoaging. However, changes of VOCs from co-PP K7726H and K9026 started to increase earlier than those from homo-PP. Co-PP resins with photoaging time up to 500~1000 h produced concentrations of acetone, butylene, tertbutyl alcohol, whose concentrations were just above the HS-GC-FID/MSD detection limit. After a longer photoaging period, sizeable amounts of acetone, butylene and tertbutyl alcohol were observed, as well as low but detectable concentrations of acetic acid, whose peak in the chromatogram was rather broad but still significant, as indicated in Figure 3 and Table 1. These results clearly showed that both the rates of the photo oxidation reactions and evaporation rates of the degradation products increased rapidly in the photoaging period. The presence of photo oxidative products from co-PP resin was earlier than that of homo-PP resin, confirming in some way the fact that homo-PP had the higher photoaging resistance than co-PP resins. It may be accounted for the backbone structure of co-PP, where the ethylene was present leading to both increased sensitivities to the secondary degradation reaction and decreased overall crystallinity in favor of the amorphous phase, allowing the better access to oxygen [40]. However, there were no significant changes of the new species of VOCs from all PP resins, indicating that photo oxidation degradation was most likely to occur in the PP fraction rather than in the EPR rubber fraction. The EPR rubber fraction of co-PP had little effect on VOCs emission during photoaging. In conclusion, co-PP was more prone to be photooxidated than homo-PP and then co-PP generated more VOCs. The differences of VOCs from co-PP between K7726H and K9026 during photoaging were significant. It seemed that the manufacturing process route influenced the formation and emission of photo oxidation degradation products, as seen in Table 1.

**Figure 4:**
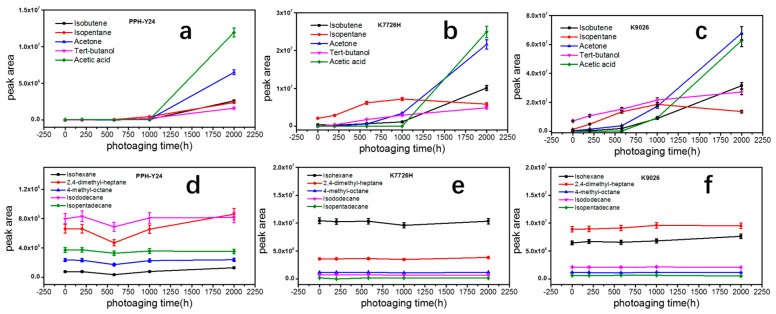
Peaks areas of different VOCs from PP resins after photoaging at different periods (**a**) and (**d**), PPH-Y24; (**b**) and (**e**), K7726H; (**c**) and (**f**), K9026).

After the photoaging of 2000 h, K9026 released acetone, isobutene, tert-butanol and acetic acid detected in the VOCs, whose concentrations were around two times higher than those of K7726H. The difference in the measured concentrations of VOCs can be attributed to different manufacturing processes and different related mechanisms. K9026 used a peroxide to degrade the molecular weight of PP during the melt pelleting process, therefore a small amount of residual peroxide would inevitably remain in PP resin and the residual peroxide as an initiator can further accelerate the photooxidative degradation of PP resin. However, K7726H did not have this phenomenon since it used the hydrogen as the molecular chain transfer agent. PP resins exposed to the photoaging of 2000 h, showed a sharp increase in amount but no difference in the species of VOCs. It showed that the continuous long-term photoaging could result in a large number of VOCs. In this case, the effect of molecular structure of PP resins on VOCs would reduce accompanying with the increase of photoaging time. During the photoaging process, the order of appearance of mainly VOCs was shown in Figure 4. Isobutylene is presented first, followed by acetone and tertbutanol and acetic acid last. It indicated that the VOCs with low boiling points may easily migrate to solid surface and then evaporate to environment. Additionally, acetone and tertbutanol were produced in the early stage of photodegradation but acetic acid was produced in the later stage. Hence, the order of presence of VOCs produced by degradation can provide a basis for the VOC formation mechanism. Moreover, some aliphatic hydrocarbons such as C_6_H_14_, C_9_H_20_, C_9_H_18_, C_12_H_26_, C_15_H_32_ and C_18_H_38_, which have been already presented in the PP without photoaging, kept stable during photoaging. It proved that these aliphatic alkane compounds were probably not products of photo degradation of PP and would not be further photooxidated into other VOCs.

Carboxylic compounds such as acetone, alcohol and carboxylic acids identified were paid attentions. Results showed that acetone and alcohol were presented in K9026 without photoaging, because of the peroxide degradation method during industrial manufacturing process. Although most residual peroxide degradation products were usually removed during the post-treatment process in industrial production, a small portion could still remain in PP, causing an amount of VOCs in ppm level.

### 2.3. Changes of Structures of PP Resin during the Accelerated Photoaging

During the photo-aging process, the surface chemical composition, molecular weight and crystallinity of the PP showed obvious changes [41,42,43,44]. Photo oxidation of PP resins produced a wide range of products. Most products were macromolecular compounds linked to the degradation of backbone, for example, chain or end-chain ketones, end-chain acids and so forth but some products were formed by processes involving several chain scissions [45]. The later products had low molecular weights and could consequently migrate out the polymers [46]. In order to understand the photo oxidation mechanism of PP resins, it is necessary to analyze the photo oxidative evolution of the PP matrix and monitor the formation of VOCs produced by photo oxidation. 

**Figure 5:**
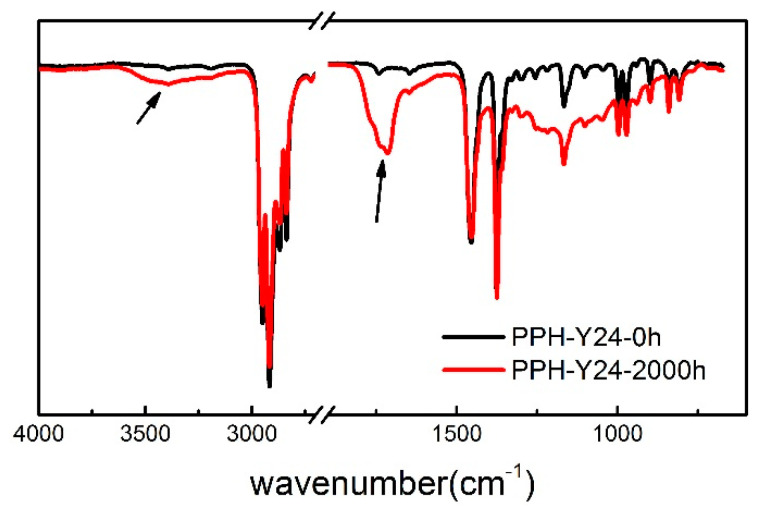
Attenuated Total Reflection Flourier transformed Infrared Spectroscopy (ATR-FTIR) of homo-PP resin irradiated for 2000 h. (the black line, without accelerated photoaging; the red line, after the accelerated photoaging).

It should be pointed out that the oxidation occurs mainly in the PP phase and less in polyethylene [45]. Therefore, the change of chemical structures of PP after photoaging was studied, using the homo-PP resin as the representative. As seen in Figure 5, FTIR spectrum of the photoaged PP was different with PP. The main changes were characteristic of the carbonylated and hydroxylated compounds, corresponding to an increase of absorbance over a broad range. These changes in the 1500–1900 cm^−1^ and 3800–3200 cm^−1^ regions corresponded to C=O and OH stretching vibration respectively [47]. The carbonyl band showed a maximum at 1720 cm^−1^ and a shoulder at 1713 cm^−1^ and the peak at 1720 cm^−1^ was related to ketones [48]. The formation of hydroxylated photoproducts led to the increase of a broad band that peaked near 3300 cm^−1^ due to hydrogen bonded by droxylated functions. The absorption at 1713 cm^−1^ is attributed to the carboxylic acids [49]. Besides, the intensities of the 1452 cm^−1^ and 1375 cm^−1^ bands increased gradually after the accelerated photoaging of 2000 h. These bands might be asymmetric and symmetric deformation of methyl groups [50]. Thus, it proved that the chain scission and photo oxidation of PP resin occurred during the accelerated photoaging process.

**Figure 6:**
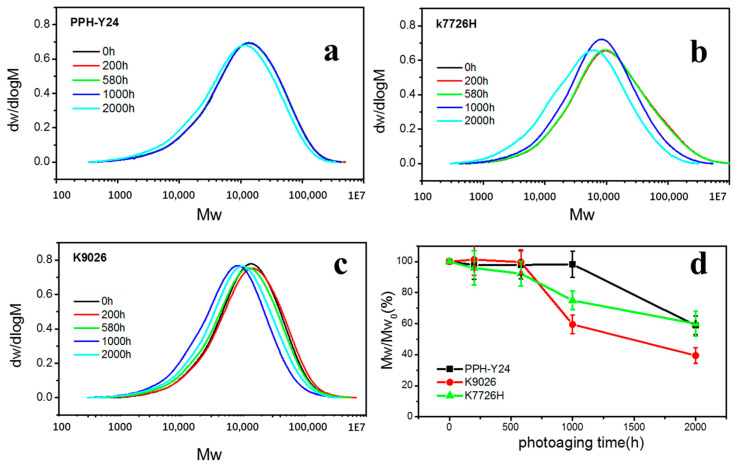
Gel Permeation Chromatography (GPC) curves (**a**–**c**) and molecular weight retentions (**d**) of PP resins with various photo degradation exposure time. ((**a**), PPH-Y24; (**b**), K7726H; (**c**), K9026).

As shown in Figure 6 and Table 2, the weights-average molecular weights (M_w_), number average molecular weight (M_n_) and Polydispersity Index (PDI) were constant when the photoaging was less than 200 h and then decreased with prolonging photoaging time. After the photoaging of 2000 h, the molecular weight of PP decreased by about 40% and the PDI of PP fluctuated about 8~12%. This showed that PP resin had undergone severe photoaging degradation, leading to a sharp decrease of molecular weight. However, the time for severe degradation of molecular chain of PP resins was different; homo-PP PPH-Y24 was about 2000 h, while co-PP K7726H and K9026 were 1000 h. This also showed that homo-PP had the better photoaging resistance than copolymer PP, which was similar to the conclusion obtained by VOCs emission behavior described earlier. As expected, irrespective of the materials, the molecular weight distribution was narrowed and the fraction of high molecular weight decreased with increasing the photoaging time.

PP morphology was also changed upon photo oxidation [51] and in some cases the crystalline order was disrupted leading to the reduction of the fractional crystallinity [52]. In other cases, however, the crystallinity was reported to increase due to chemi-crystallization during photo oxidation [53]. The most important practical consequence of chemi-crystallization was the spontaneous formation of surface cracks caused by contraction of the surface layers [53]. The presence of surface cracks would not only cause a serious deterioration of the mechanical properties of the products after short-term exposures [54] but also accelerate the VOCs released from the interior of PP matrix to the external environment.

**Figure 7:**
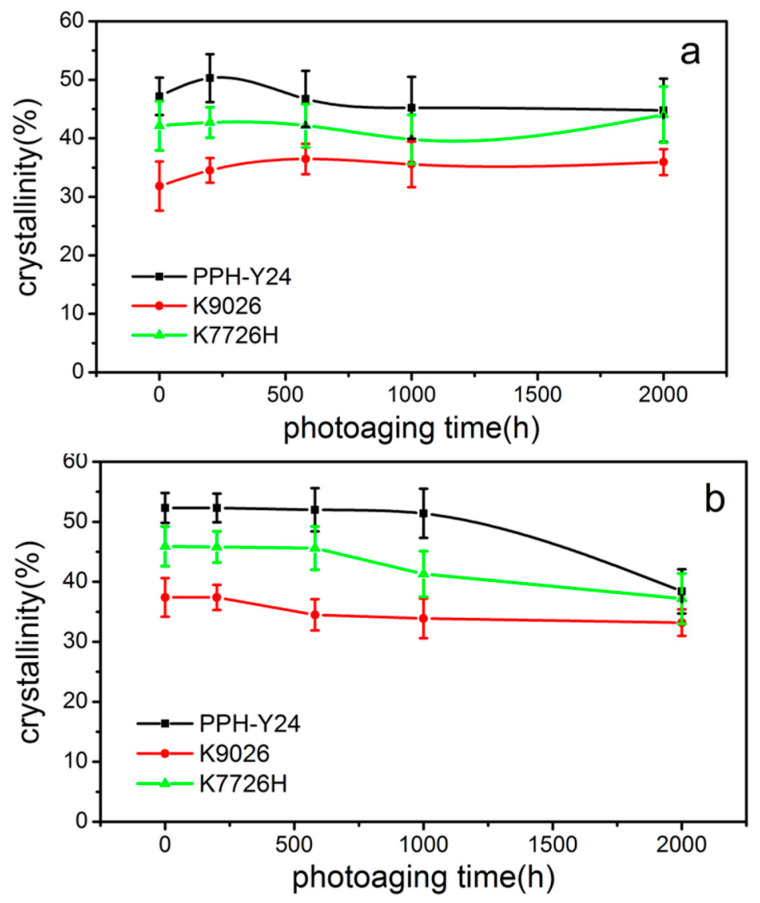
Crystallinity of PP resins during different photoaging time ((**a**) crystallinity from the first heating curve; (**b**) crystallinity from the second heating curve).

As shown in Figure 7, the crystallinity from both the first and second heating curves were different with the increasement of photoaging time. It can be seen that with the prolongation of photo aging time, the crystallinity of PP resins from the first heating curve firstly increased and then decreased, at last increased again. It was seemed that the crystallinity of PP was promoted because of annealing at the higher temperature and then decreased because of the chemi-crystallization by the photooxidation. However, the crystallinity of PP resins from the second heating curve firstly almost unchanged and then decreased. The second run contained information about the content of defects (i.e., carbonyl groups) caused by weathering. Therefore, these chemical defects could reduce the crystallinity of the PP resin.

**Figure 8:**
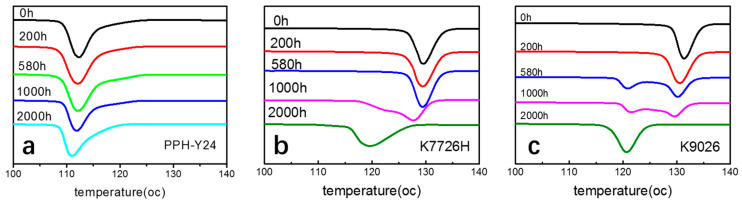
Differential scanning calorimetry (DSC) thermograms for the non-isothermal crystallization of PP resins after photoaging at different photoaging time. ((**a**), PPH-Y24; (**b**), K7726H; (**c**), K9026; heat flow endo up).

As shown in Figure 8, it can be found that the DSC thermograms for the non-isothermal crystallization of three PP resins upon different photoaging time are quite different. The non-isothermal crystallization thermogram of homo-PP has a single peak. However, the DSC thermograms for the non-isothermal crystallization of co-PP K7726H have double exotherms when the photoaging time was 1000 h and longer, with a peak or shoulder situated on the lower temperature side of thermograms. The intensity of the subsidiary peak is higher upon the photoaging time of 1000 h and longer. K9026 also has double exothermers, similar to K7726H but the photoaging time of similar behavior is shifted to earlier. The crystallization of photoaged PP would be mainly resulted from the chain scission, build-up of carbonyl and from the presence of other impurity groups. The former favored the crystallization whereas the later rendered crystallization [53].

**Figure 9:**
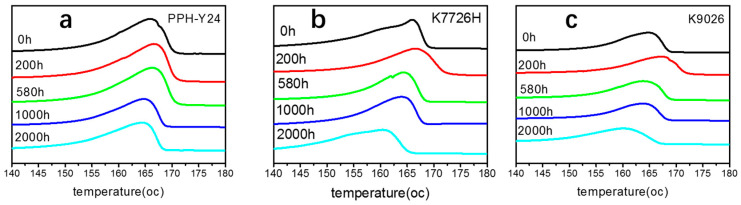
DSC 1^st^ melting thermograms of PP resins with various photoaging time. ((**a**) PPH-Y24; (**b**) K7726H; (**c**), K9026; heat flow endo up).

As shown in Figure 9, the melting temperature increases firstly and then decreases with increasing photoaging time. The increase of T_m_ at the initial stage of photoaging indicated that the thickness of lamellae increased due to the simple an effect of rising sample temperature. It showed that the photo aging can increase the crystallinity of PP to a certain extent. Subsequently, due to the photo degradation, the crystalline region of PP was destroyed and the oxidative reactions on the crystal surface increased the surface free energy of the crystals [55]. Therefore, it led to the decrease of T_m_. Therefore, the photo oxidation of PP led to the imperfect crystal and then accelerated VOCs emission due to the lower T_m_ and the wider crystallization range.

### 2.4. Mechanism of VOC Formation from PP Resin

In order to explain the nature and the relative proportions of VOCs from different stages, an interpretation in terms of mechanisms is required. The most probable mechanisms for the main VOCs have been arbitrarily proposed based on the full-processing manufacturing route of neat PP resin such as the polymerization processing, the extrusion pelleting process and the photo oxidative degradation, as well as the properties of VOCs such as the polarity or the boiling point and also on basis of published experimental evidences [37,38,39].

The most VOCs including aliphatic hydrocarbons, such as 2-methyl-pentane (C_6_H_14_), 2,4-dimethyl-heptane (C_9_H_20_), 4-methyl-octane (C_9_H_20_), 2,4,6-trimethyl-nonane (C_12_H_26_) and 4,6-dimethyl-decane (C_12_H_26_) from neat PP resin as listed in Table 1 was generated from both the polymerization processing and the pelleting process of twin screw extruder in the industrial manufacturing process.

On the one hand, the molecular weight of PP is reduced by adding hydrogen in the polymerization, which is the main control technology for the PP molecular weight, because transfer with hydrogen is the most important chain termination process under normal polymerization condition with Ziegler-Natta heterogeneous catalysts. As the molecular chain transfer agent, hydrogen can quickly combine with multiple active centers of Ti of the catalyst to terminate the PP molecular chain growth and can thereby effectively generate oligomers with various molecular weights, such as C_3_H_8_, C_6_H_14_, C_9_H_20_, C_12_H_26_, C_15_H_32_, C_18_H_38_, C_21_H_44_ and so forth. However, only some VOCs ranged from C_6_ to C_18_ can migrate out of the solid PP matrix as a consequence of their low boiling points, with exception of C_3_H_8_ devolatilized by a special treatment and alkanes compounds above C_18_ with high boiling points.

**Scheme 1:**
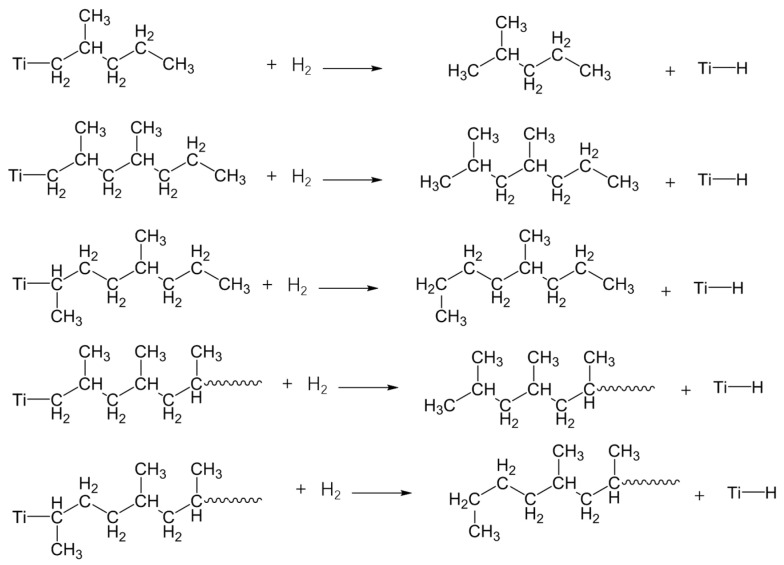
The mechanistic scheme presenting the formation of the main VOCs by polymerization from neat PP resin.

In addition, the insertion of PP during polymerization in the metal-carbon bond may take place in two different ways; 1, 2-insertion and 2, 1-insertion. It is unambiguously proved, by the chain-end analysis, that the 1, 2-insertion mode is operative in the isospecific polymerization of olefins and also of styrene [56,57]. Region-irregular 2,1-insertion is particularly prevalent at the start of chain growth for PP prepared in the presence of hydrogen, because 2,1-insertion, followed by the usual 1,2-insertion into a Ti-H bond has a far great probability than 2,1-insertion into Ti-C [58]. Therefore, as shown in Scheme 1, various insertions of PP would lead to the variety of VOCs with different molecular structures. For example, the formation of 2-methyl-pentane (C_6_H_14_), 2,4-dimethyl-heptane (C_9_H_20_) and 2,4,6-trimethyl-nonane (C_12_H_26_) are arise from the region-regular 1,2-insertion, while 4-methyl-octane (C_9_H_20_) and 4,6-dimethyl-decane (C_12_H_26_) are resulted from the region-irregular 2,1-insertion in the polymerization.

**Scheme 2:**
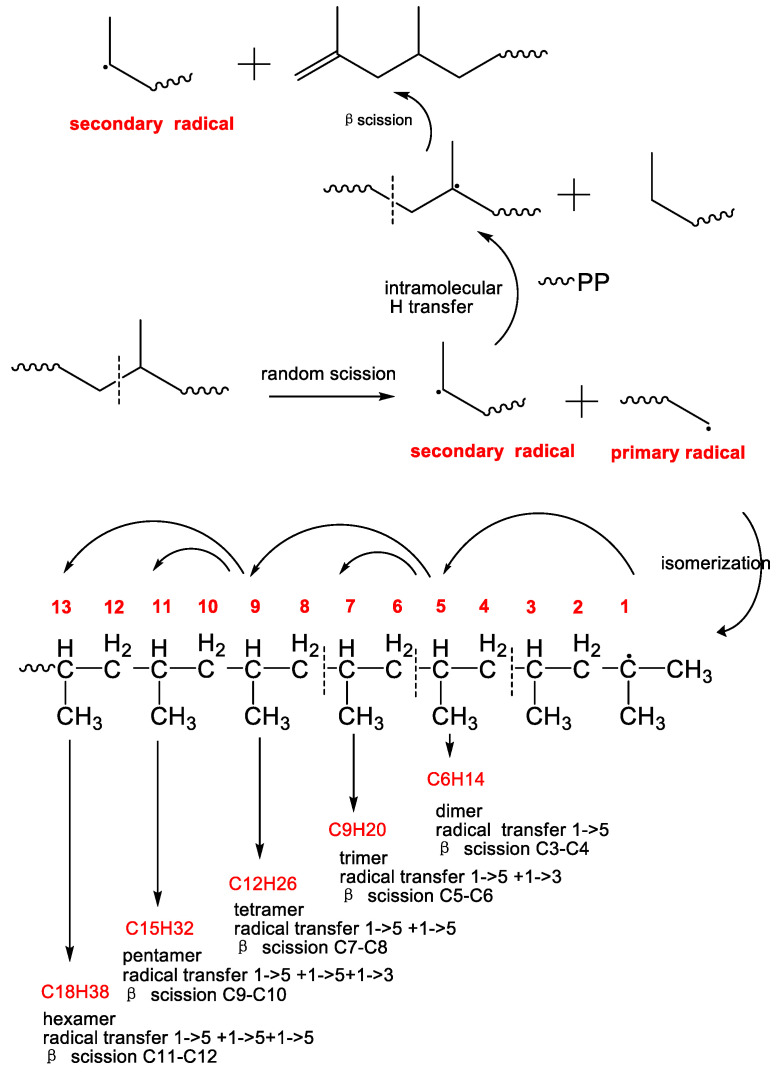
The mechanistic scheme presenting the formation of the main VOCs by extrusion pelleting from neat PP resin.

On the other hand, the VOCs of aliphatic hydrocarbons could be also produced from the pelleting process. PP powder is usually pelleted by the twin-screw extruder. Therefore, several VOCs could be produced by the thermomechanical degradation because of the oxygen-free state in the barrel of the twin-screw extruder with the continuous nitrogen protection in industrial plant. After the C-C bond scission into primary radical and secondary radical [59], tertiary radicals were formed via rearrangement reactions. The subsequent β-scission of secondary radical leads to the formation of PP chain containing a terminated double bond and the chain carrier secondary radical where the cycle was continued to generate PP chain-end with double bond and secondary radical [60]. However, according to the above mechanism, it was impossible to generate these detected aliphatic hydrocarbons from PP resins. Therefore, the above mechanism should be excluded in the formation of VOCs from PP. Instead, as shown in Scheme 2, the tertiary radical isomerized by the primary radical and via intramolecular hydrogen transfer to the 5th, 7th, 9th and 13th carbon atom in the primary macroradical via six-membered ring intermediates were preferred, produced various volatile oligomers range from C_6_ to C_18_ [61]. Due the high alkane concentration in contrast to the alkene, transfer reactions seemed to play a major role. Therefore, it seemed reasonable that volatile alkane molecules were formed from the primary radicals via intramolecular hydrogen transfer reactions. The chain carrier secondary radical did not undergo transfer reactions.

According to the foregoing analysis, the formation of VOCs from photoaged PP resins mainly came from neat PP resin and photo oxidation degradation of PP resin. Towards the VOCs caused by photoaging of PP resin, it is necessary to start from the species and proportions of VOCs combined with the photo-oxidative degradation mechanism of PP to reveal the mechanism of VOCs formation. The formation of most new emerged volatile degradation products from photoaged PP was resulted from the photo oxidation mechanism, which has been described in many prior publications about PP degradation [37,39]. Identification and quantification of VOCs could bring valuable information about the prevailing mechanisms of PP photo oxidation, in consistent with their precursory macromolecular oxidation products [36,37,38,39].

In the foregoing, FTIR and GPC analysis showed that the photo aging caused a decrease of molecular weight and the formation of oxygen-containing chemical groups. In addition, DSC analysis showed that the crystal region was damaged in varied degrees, leading to the formation of cracks on the surface [53]. This made the migration of VOCs from the amorphous region to the sample surface much easier. Therefore, VOC release was accelerated after photo aging. Besides, the generation of VOCs could be accompanied by the macromolecular chain scission and oxidation products, which would provide the relevant experimental basis for revealing the formation mechanism of VOCs.

**Scheme 3:**
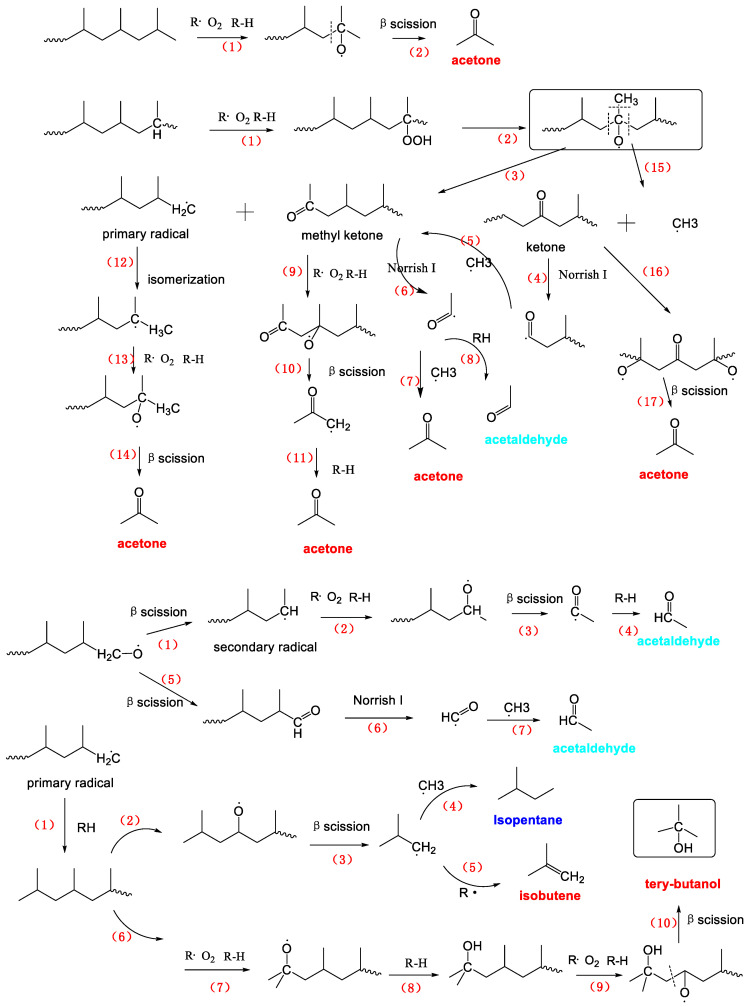
The mechanistic scheme presenting the formation of the main VOCs from the photo-aged PP resin.

There were several main steps involved in the formation of many volatile products emanating from PP photoaging degradation (Scheme 3). The initial attack of the PP macromolecule involves the readily abstracted hydrogen atom of a tertiary carbon atom, leading to the formation of a peroxide and, which later decomposes to either alkoxy radical and methyl ketone or to a tertiary alcohol. This initial process of the tertiary carbon resulted in a large content of macromolecular oxidation products, which was consistent with our FTIR analysis as well as previous literatures [43,44,45]. The alkoxy radical could lead to two different oxidation products; a primary radical and a tertiary carbon methyl ketone. The methyl ketone route involved the cleavage of the macromolecular chain, which provided one of the two cleavage steps necessary to produce small molecule products. Besides, the volatile degradation products of PP is related to the fate of the primary radical that arisen at the other chain end, simultaneously with the methyl ketone formation as shown in Scheme 3. This primary radical could abstract a hydrogen atom or couple with free radicals to yield a chain end methyl group or undergo a disproportionation with another radical to form a chain end double bond or else react with oxygen to form a peroxide which can subsequently lead to several other oxidation products including a primary alcohol or an aldehyde. Moreover, the oxidation of primary radical competes with the isomerization by intramolecular hydrogen transfer that gave more stable tertiary radical. Oxidation of the tertiary radical led to formation of acetone and primary radical by β scission. The primary radical was involved in the further reaction, producing acetone and acids. The propagation of this reaction resulted finally in a notable decrease of the chain length, which is consistent with GPC results (Figure 6). It should be noted that both β scission [37,39] of alkoxy radicals and Norrish tape I [47] reactions of ketones were probably the main generation routes for VOCs formation. Consequently, some radicals including the carbonyl radical, the hydrogen radical and the methyl radical formed during the photo oxidation process can spontaneously combine with other small weight radicals to produce various volatile compounds mentioned in this paper. Next, the main VOCs formation process was described in detail.

The formation of acetone (C_3_H_6_O) would be formed from the primary radical, the secondary radical, the tertiary radical and the methyl ketone by five pathways as already discussed in Scheme 3. First, a tertiary radical can be produced by the oxidation of PP chain or the isomerization of primary radical. The oxidation and then β scission of the tertiary radical can directly lead to form acetone. Secondly, the carbonyl radical, produced by Norrish tape I [46] reactions of ketones products, combined with RH (PP chain) and methyl radicals can form acetone.

As shown in Scheme 3, isobutene (C_4_H_8_) was resulted from reactions initiated by the radical attack on the second-from-the-end tertiary carbon atom in the methyl-terminated polymer chain end.

Robert Bernstein et al. [37] have found that tertiary alcohols were a major product on basis of results on the macromolecular products of PP radiation oxidation. Formation of a tertiary alcohol was not, however, involved of any chain scissions. But the formation of volatile products for instance tertiary alcohol would necessarily require additional chain scissions chemistry on both sides of the tertiary alcohol sites [37]. Thus, tertiary butanol (C_4_H_10_O) would be formed by β reactions of alkoxy radical with the tertiary carbon site near the macromolecular chain end which has been already occupied by an alcohol group shown in Scheme 2.

The isopentane (C_5_H_12_) could be generated through the radical-radical recombination reaction, in which one of the two radicals was (·CH_3_) and the other was the isobutyl radical (·CH_2_CH(CH_3_)CH_3_) which was produced by β scission of alkoxy radical with the tertiary carbon site near the macromolecular chain end.

Moreover, the secondary atom radical can possibly abstract a hydrogen atom to yield a chain end methyl group, including alkane range from C_6_H_14_ to C_18_H_38_ shown in Scheme 3. The amounts of these alkane VOCs, such as C_6_H_14_, C_9_H_20_, C_12_H_26_, C_15_H_32_ and C_18_H_38_, identified from the neat PP and photoaged PP have little changes. Results indicated that all alkane compounds (except for C_5_H_12_) were not produced by this pathway from the photo aging process.

Acetic acid (C_2_H_6_O_2_) is hydrogen abstraction from the secondary carbon adjacent to a methyl ketone, to yield a relatively favorable radical (due to the conjugation with the carbonyl group) [39]. Eventual coupling action of existed hydroxyl radicals gave acetic acid. As the second possible route, the alkoxy radical from primary radical can yield the secondary radical, which should produce PP with chain end aldehyde and yield polymer acid. The first tertiary carbon atom near the hydroxyl carbon was oxidized to alkoxy radical, which was finally yielded acetic acid by β scission.

Acetaldehyde (C_2_H_4_O) yielded from primary radical, secondary radical and methyl ketone was similar to acetone formation exception of isomerization of primary radical.

It is found that the acetone would be relatively more abundant than other volatile compounds. This is mainly due to its low boiling point and the multi-path formation mechanism. Furthermore, acetic acid is hardly presented in the early stage of photoaging degradation of PP but it increases sharply in the late stage of photoaging. This may be mainly due to the higher boiling point and the relatively long process of acetic acid production, which required the continuous photoaging to accumulate a certain time before the further oxidation.

## 3. Materials and Methods

### 3.1. Materials and Preparation

Three commercial neat PP resins in pellets form with the similar MFR were chosen in this work. For specially, three grades PP resins, PPH-Y24, K7726H and K9026, used different manufacturing processes were provided by SINOPEC Inc., Beijing, China. PPH-Y24 was isotactic polypropylene (iPP). While, both K7726H and K9026 were impact co-PP consisting of PP fraction and ethylene-propylene rubber (EPR) fraction. Impact co-PP was a reactor alloy produced by the multi-reactor process in which PP was homopolymerized in the first reactor and then EPR was copolymerized by adding ethylene into the following reactor(s). In addition, the PP resins K9026 was manufactured by peroxide degradation method [62,63], however, both PPH-Y24 and K7726H were produced through hydrogen regulation method (direct polymerization using novel catalysts and addition hydrogen) [64,65]. The information of three grades of PP resins used in the study were given in Table 3.

Accelerated photoaging test of PP resins was performed in the Q-SUN XE-3-HDS Xenon test chamber (Q-Labs Corp., Cleveland, Ohio, OH, USA). For specially, the transparent 10 mL headspace vials containing PP samples at the weight of 1 ± 0.01 g were sealed with caps. The vials containing the PP pellet were then placed flat directly in the test chamber for photoaging test. In order to prevent the air leakage caused by the aging of the bottle cap, vial caps were wrapped with aluminum foils. Weathering condition was set at the temperature of 38 °C the relative humidity (RH) of 50 RH% and the irradiance of 0.51 W/(m^2^ nm), following the modified ISO 4892–2 standard.

### 3.2. Characterizations

VOCs from PP resins was analyzed by HS-GC-FID/MS, which was carried out by automatic headspace sampler Agilent 7697A in combination with Agilent 7890B series GC system with FID, equipped with mass spectrometer Agilent 5977 (Palo Alto, California, CA, USA). HS-GC-FID/MS analysis consisted of two steps. First, the specimen with a weight of 1 ± 0.01 g was placed in a 10 mL headspace vial that had a gas volume above it and the vial was closed and then thermostatted at a constant temperature until equilibrium was reached between the two phases. Then an aliquot of the vial’s gas phase was introduced into the carrier gas stream which carried it into the column, where it was analyzed. A modified VDA 277 method, the test standard of German automotive industry association, was referred for the VOCs analysis by HS extraction from samples. In particular, the VOCs were identified by the Synchronous Online FID/MSD dual detector. Triplicates were used for each measurement.

The molecular mass distribution (MWD) of samples was analyzed by Gel Permeation Chromatography (GPC 220, Varian, Palo Alto, California, CA, USA). Each sample was dissolved in 1,2,4-trichlorobenzene. GPC was run at the temperature of 150 °C Polystyrene standards with the narrow MWD was used for calibration.

ATR-FTIR was measured in a Thermo Scientific (Nicolet 670, Waltham, MA, USA) FT-IR spectrometer with a single diamond reflection ATR accessory. The FTIR spectra were recorded with a 4 cm^−1^ spectral solution between 4000 cm^−1^ and 750 cm^−1^ for the average of 32 scans. Triplicates were used for each measurement.

DSC measurements were carried out on a Perkin-Elmer (Waltham, MA, USA) Pyris Diamond DSC instrument with a nitrogen circulation under N_2_ gas flow (20 mL/min). The experiments were performed in a heating/cooling program from 30 °C to 220 °C at a rate of 10 °C/min. Samples of triplicates with the weight of 5~8 mg were performed. The melting temperature (T_m_) and the crystallization temperature (T_c_) of PP resins were measured from the first heating and cooling stage, respectively. The crystallinity (Xc) was calculated from the melting enthalpy of DSC first heating curve. Xc = ΔH_m_/ΔH_0,_ ΔH_0_ = 209 J/g [66].

## 4. Conclusions

HS-GC-FID/MSD was successfully used to detect, identify and quantify a large number of VOCs generated during manufacturing and accelerated photoaging of neat homo-PP and co-PP resins. More than 40 species of VOCs were clearly evidenced. Most VOCs were major aliphatic hydrocarbons (up to C_18_) and small amounts of ethanol, acetone and tertbutanol. All VOCs were formed from polymerization and pelleting during the manufacturing of PP resin. The species and amounts of VOCs from homo-PP resin were lower than those of co-PP resin. Moreover, the VOCs of PP resins with different manufacturing processes were significantly different. The species, amounts and odors of VOCs of PP prepared by the hydrogen regulation method were lower than those of PP prepared by the peroxide degradation method.

Photoaging increased the VOCs emission from PP resins. Moreover, the species and amounts of VOCs increased with extending the photoaging time. The VOCs produced by the photo oxidation were mainly alkane, alkene and volatile compounds with oxygen-containing group including aldehydes, ketones, alcohols and acid compounds. The VOCs from PP resins with various molecular structures and manufacturing processes were affected differently in response to the photoaging. The amount of VOCs from homo-PP was the lowest, followed by that from co-PP with hydrogen regulation method and that from co-PP with peroxide degradation method was the highest. Moreover, towards VOCs from co-PP, the oxidation photoproducts were from the PP fraction rather the EPR rubber fraction. Besides, during photoaging, PP underwent the changes of surface chemical composition, molecular weight, molecular weight distribution and crystalline form. The chain scission and photo oxidation of PP resin occurred during the accelerated photoaging process. Both T_c_ and T_m_ of PP decreased and the crystallization of PP was also changed with the photo aging. The surface cracks accelerated the migration of VOCs from the solid matrix to the surface and then to the external environment.

The formation of VOCs from manufacturing and photoaging were different. The former involved in coordination polymerization and thermomechanical free radical degradation. However, the latter referred to the photo oxidation. β scission of alkoxy radical and Norrish tape I reactions of ketones via intermediate transition were probably the main generation routes for the formation of VOCs. The VOCs may be produced from multiple pathways including the primary radical, the secondary radical, the tertiary radical as well as the methyl ketone.

## Figures and Tables

**Table 1:** Changes of VOCs of neat polypropylene (PP) resins during the accelerated photoaging monitored by Headspace extraction-Gas Chromatography/Mass Spectroscopy (HS-GC/MS).

No	Retention Time (min)	Molecular Formula	Compounds Name	CAS Number	Peak Area (×10^5^) PPH-Y24					Peak Area (×10^5^) K9026					Peak Area (×10^5^) K7726H				
					0 h	200 h	580 h	1000 h	2000 h	0 h	200 h	580 h	1000 h	2000 h	0 h	200 h	580 h	1000 h	2000 h
1	2.07	C_4_H_8_	Butene	25167–67–3	/	0.20 ± 0.01	0.30 ± 0.02	0.84 ± 0.03	26.17 ± 1.44	0.93 ± 0.04	5.49 ± 0.21	20.31 ± 1.05	89.49 ± 4.07	315.61 ± 7.25	4.03 ± 0.23	3.09 ± 0.11	6.91 ± 0.33	11.77 ± 0.44	101.38 ± 5.00
2	2.12	C_5_H_12_	Isopentane	78–78–4	0.10 ± 0.00	0.63 ± 0.03	1.02 ± 0.06	4.57 ± 0.25	23.86 ± 1.31	13.86 ± 0.72	50.01 ± 2.14	135.00 ± 6.55	186.75 ± 9.85	137.42 ± 6.01	21.16 ± 1.23	28.96 ± 1.54	62.00 ± 3.23	71.97 ± 3.21	58.61 ± 2.32
3	2.23	C_6_H_14_	2-Methylpentane	107–83–5	0.77 ± 0.02	0.77 ± 0.03	0.47 ± 0.02	0.79 ± 0.02	1.30 ± 0.08	64.59 ± 3.25	67.17 ± 3.41	65.88 ± 3.02	68.25 ± 3.54	76.47 ± 3.52	104.28 ± 5.54	102.73 ± 5.26	103.38 ± 5.12	103.15 ± 5.11	103.53 ± 5.02
4	2.29	C_6_H_14_	Hexane	110–54–3	/	/	/	/	/	/	/	/	/	/	11.48 ± 0.55	11.05 ± 0.81	11.08 ± 0.35	10. 45 ± 0.42	15. 14 ± 0.49
5	2.43	C_6_H_12_	2-methyl-1-pentene	763–29–1	/	0.20 ± 0.01	0.19 ± 0.01	0.54 ± 0.04	1.13 ± 0.07	4.44 ± 0.28	5.37 ± 0.21	6.36 ± 0.22	6.78 ± 0.41	7.56 ± 0.32	/	/	/	/	/
6	2.65	C_2_H_4_O	Acetaldehyde	75–07–0	0.07 ± 0.00	1.41 ± 0.08	0.35 ± 0.03	0.85 ± 0.04	5.90 ± 0.33	3.90 ± 0.24	5.04 ± 0.30	5.94 ± 0.25	16.68 ± 0.48	24.84 ± 1.62	0.56 ± 0.02	0.93 ± 0.03	4.25 ± 0.27	5.87 ± 0.20	9.21 ± 0.62
7	3.31	C_9_H_2_0	2,4-dimethyl-heptane	2213–23–2	6.61 ± 0.21	6.62 ± 0.28	6.11 ± 0.40	6.56 ± 0.32	8.61 ± 0.47	89.07 ± 4.55	89.61 ± 4.81	91.32 ± 4.01	96.12 ± 5.02	95.31 ± 5.02	36.23 ± 1.35	36.21 ± 1.99	36.71 ± 1.87	35.04 ± 1.55	38.65 ± 1.58
8	3.54	C_3_H_6_O	Acetone	67–64–1	/	0.25 ± 0.01	0.38 ± 0.02	1.16 ± 0.07	64.93 ± 3.33	4.53 ± 0.28	15.27 ± 0.74	39.39 ± 1.29	172.98 ± 8.78	678.36 ± 36.01	/	0.70 ± 0.07	6.24 ± 0.40	34.28 ± 1.44	216.33 ± 11.81
9	3.86	C_9_H_20_	4-Methyloctane	2216–34–4	2.36 ± 0.18	2.33 ± 0.15	2.25 ± 0.12	2.28 ± 0.14	2.40 ± 0.14	11.55 ± 0.51	11.58 ± 0.55	11.34 ± 0.39	12.09 ± 0.44	11.55 ± 0.49	11.83 ± 0.55	11.85 ± 0.66	11.98 ± 0.71	11.19 ± 0.51	11.99 ± 0.55
10	4.22	C_9_H_18_	2,4-Dimethyl-1-heptene	19549–87–2	0.20 ± 0.01	0.20 ± 0.01	/	0.19 ± 0.01	0.41 ± 0.04	18.84 ± 0.97	19.68 ± 0.94	19.05 ± 0.88	15.33 ± 0.62	6.69 ± 0.28	1.18 ± 0.04	1.16 ± 0.05	1.10 ± 0.04	0.96 ± 0.04	0.89 ± 0.03
11	4.52	C_4_H_9_OH	tert-Butanol	75–65–0	/	0.25 ± 0.01	0.49 ± 0.02	2.22 ± 0.12	16.05 ± 0.92	71.97 ± 3.66	108.03 ± 5.01	153.54 ± 7.04	216.39 ± 11.02	272.22 ± 12.25	/	3.86 ± 0.12	17.89 ± 0.78	29.38 ± 1.36	49.08 ± 2.57
12	5.09	C_2_H_5_OH	Ethanol	64–17–5	/	/	/	/	/	1.48 ± 0.05	1.55 ± 0.06	1.61 ± 0.05	1.53 ± 0.05	1.55 ± 0.08	2.86 ± 0.12	2.87 ± 0.14	2.95 ± 0.14	2.97 ± 0.10	2.94 ± 0.12
13	6.38	C_12_H_26_	/	/	7.99 ± 0.42	8.32 ± 0.41	8.17 ± 0.44	8.09 ± 0.47	8.16 ± 0.47	21.4 ± 1.62	21.09 ± 1.00	21.09 ± 1.02	21.63 ± 1.07	20.82 ± 1.12	7.42 ± 0.41	7.47 ± 0.33	7.55 ± 0.28	6.81 ± 0.25	6.97 ± 0.41
14	6.46	C_12_H_26_	/	/	3.40 ± 0.11	3.43 ± 0.01	3.94 ± 0.21	3.36 ± 0.14	3.83 ± 0.23	2.70 ± 0.14	2.70 ± 0.12	2.64 ± 0.10	3.84 ± 0.21	5.61 ± 0.26	1.73 ± 0.08	1.75 ± 0.08	1.98 ± 0.12	2.04 ± 0.09	2.93 ± 0.14
15	7.24	C_12_H_26_	/	/	4.03 ± 0.10	4.04 ± 0.20	4.55 ± 0.25	3.90 ± 0.23	1.79 ± 0.07	4.38 ± 0.20	4.32 ± 0.25	4.47 ± 0.20	1.23 ± 0.05	1.50 ± 0.04	4.20 ± 0.24	4.21 ± 0.22	4.22 ± 0.22	3.97 ± 0.21	1.28 ± 0.05
16	7.35	C_12_H_26_	/	/	1.74 ± 0.08	1.75 ± 0.18	2.01 ± 0.12	1.69 ± 0.04	1.79 ± 0.06	1.08 ± 0.04	1.11 ± 0.05	0.99 ± 0.04	4.59 ± 0.14	2.94 ± 0.16	0.97 ± 0.08	0.99 ± 0.04	0.99 ± 0.04	0.95 ± 0.04	0.16 ± 0.01
17	8.37	C_4_H_1_0O	1-Butanol	71–36–3	/	/	/	/	4.50 ± 0.24	0.33 ± 0.01	0.30 ± 0.04	0.33 ± 0.01	3.54 ± 0.19	8.61 ± 0.65	1.91 ± 0.14	1.89 ± 0.07	1.51 ± 0.07	1.87 ± 0.07	4.40 ± 0.22
18	9.49	C_15_H_32_	/	/	3.72 ± 0.17	3.75 ± 0.22	4.27 ± 0.21	3.58 ± 0.11	3.51 ± 0.15	6.39 ± 0.34	6.36 ± 0.33	6.45 ± 0.19	6.69 ± 0.42	5.49 ± 0.21	2.14 ± 0.12	2.14 ± 0.14	2.13 ± 0.18	2.01 ± 0.12	1.75 ± 0.07
19	9.57	C_5_H_8_O_2_	2,4-Pentanedione	123–54–6	/	/	/	/	15.24 ± 0.74	/	/	/	/	/	/	/	/	/	/
20	10.27	C_15_H_32_	/	/	1.68 ± 0.07	1.70 ± 0.14	1.96 ± 0.14	1.63 ± 0.07	1.78 ± 0.08	1.44 ± 0.08	1.44 ± 0.08	1.44 ± 0.05	1.65 ± 0.05	1.71 ± 0.07	1.22 ± 0.07	1.22 ± 0.08	0.16 ± 0.01	1.18 ± 0.05	1.28 ± 0.05
21	11.99	C_18_H_38_	/	/	1.33 ± 0.05	1.33 ± 0.08	1.52 ± 0.14	1.26 ± 0.09	1.34 ± 0.09	2.25 ± 0.10	2.25 ± 0.12	2.13 ± 0.14	2.34 ± 0.14	2.64 ± 0.11	0.66 ± 0.04	0.67 ± 0.04	0.64 ± 0.05	0.62 ± 0.08	0.69 ± 0.02
22	12.64	CH_3_COOH	Acetic acid	64–19–7	0.60 ± 0.04	0.58 ± 0.05	0.62 ± 0.03	0.55 ± 0.04	119.44 ± 5.77	0.45 ± 0.03	0.42 ± 0.01	1.53 ± 0.07	93.63 ± 3.58	626.94 ± 32.01	0.38 ± 0.02	0.38 ± 0.03	0.38 ± 0.01	0.41 ± 0.01	249.38 ± 12.08

**Table 2:** The molecular weights Mw and Mn and the polydispersity index for all PP resins.

PP Grades	PPH-Y24			K7726H			K9026		
Photoaging Time (h)	Mn (×10^4^)	Mw (×10^4^)	Polydispersity Index	Mn (×10^4^)	Mw (×10^4^)	Polydispersity Index	Mn (×10^4^)	Mw (×10^4^)	Polydispersity Index
0	3.3	23.6	7.2	4.5	22.0	4.9	3.6	30.4	8.4
200	3.2	23.1	7.2	4.3	21.1	4.9	3.6	30.8	8.6
580	3.2	23.1	7.2	3.6	20.3	5.6	3.6	30.3	8.4
1000	3.1	23.2	7.5	2.8	16.5	5.9	2.8	18.1	6.5
2000	2.1	13.9	6.6	2.4	13.2	5.5	1.6	12.0	7.5

**Table 3:** Composition and properties of three grades of PP resins used in the study.

Resin Type	Grade	MFR (g/10 min)	Comonomer	Molecular Weight Modifier	Stage of Loading
homo-PP	PPH-Y24	26	/	H_2_	polymerization
impact co-PP	K7726H	26	ethylene	H_2_	polymerization
impact co-PP	K9026	26	ethylene	peroxide	extrusion pelleting
